# Climate change, woodpeckers, and forests: Current trends and future modeling needs

**DOI:** 10.1002/ece3.4876

**Published:** 2019-02-05

**Authors:** Eric S. Walsh, Kerri T. Vierling, Eva Strand, Kristina Bartowitz, Tara W. Hudiburg

**Affiliations:** ^1^ Forest, Rangeland, and Fire Sciences Department University of Idaho Moscow Idaho; ^2^ Department of Fish and Wildlife Sciences University of Idaho Moscow Idaho

**Keywords:** climate change, forest ecosystems, interdisciplinary modeling, review, woodpeckers

## Abstract

The structure and composition of forest ecosystems are expected to shift with climate‐induced changes in precipitation, temperature, fire, carbon mitigation strategies, and biological disturbance. These factors are likely to have biodiversity implications. However, climate‐driven forest ecosystem models used to predict changes to forest structure and composition are not coupled to models used to predict changes to biodiversity. We proposed integrating woodpecker response (biodiversity indicator) with forest ecosystem models. Woodpeckers are a good indicator species of forest ecosystem dynamics, because they are ecologically constrained by landscape‐scale forest components, such as composition, structure, disturbance regimes, and management activities. In addition, they are correlated with forest avifauna community diversity. In this study, we explore integrating woodpecker and forest ecosystem climate models. We review climate–woodpecker models and compare the predicted responses to observed climate‐induced changes. We identify inconsistencies between observed and predicted responses, explore the modeling causes, and identify the models pertinent to integration that address the inconsistencies. We found that predictions in the short term are not in agreement with observed trends for 7 of 15 evaluated species. Because niche constraints associated with woodpeckers are a result of complex interactions between climate, vegetation, and disturbance, we hypothesize that the lack of adequate representation of these processes in the current broad‐scale climate–woodpecker models results in model–data mismatch. As a first step toward improvement, we suggest a conceptual model of climate–woodpecker–forest modeling for integration. The integration model provides climate‐driven forest ecosystem modeling with a measure of biodiversity while retaining the feedback between climate and vegetation in woodpecker climate change modeling.

## 1 INTRODUCTION

As global atmospheric CO_2_ has increased, the United States has warmed 0.7°C–1.1°C, with most of the warming occurring since 1970 (Walsh et al., 2014) impacting forest ecosystems (Anderson‐Teixeira et al., 2013). Globally, forests provide many ecosystem services, including sequestration of ~30% of global annual anthropogenic CO_2_ emissions (Pan et al., 2011) and habitat for 77% of the global avifauna (BirdLife International, 2017). Climate warming and changing precipitation regimes have impacted forest ecosystem structure and function (Anderson‐Teixeira et al., 2013), including North American avifauna populations (Prince & Zuckerberg, 2015; Tingley, Koo, Moritz, Rush, & Beissinger, 2012). Moreover, predictions indicate that more than half of the forested land cover of North America will experience future climates that differ from historical growing conditions (Charney et al., 2016) with obvious implications for preservation of wildlife biodiversity (Langdon & Lawler, 2015), since forest composition and structure are integral to biodiversity (McElhinny, Gibbons, Brack, & Bauhus, 2005).

The structure and composition of forest ecosystems are expected to shift with climate‐induced changes in precipitation, temperature (Lenihan, Bachelet, Neilson, & Drapek, 2008), fire (Abatzoglou & Williams, 2016), carbon mitigation strategies (Hudiburg, Luyssaert, Thornton, & Law, 2013; Law et al., 2018; Law, Hudiburg, & Luyssaert, 2013), and biological disturbances (Weed, Ayres, & Hicke, 2013). Specifically, climate change is expected to cause declines in tree species occurrence (Coops & Waring, 2011a), shifts in carbon stocks (Lenihan et al., 2008), increases in forest mortality events (Allen et al., 2010; McDowell & Allen, 2015), and increases in burned area (Rogers et al., 2011J). These changes will affect avifauna habitat. For example, moderate‐ to high‐severity fires can create open forests, adequate snag density, and minimal midstory vegetation necessary for some woodpecker habitat (Hoyt & Hannon, 2002; Vierling, Lentile, & Nielsen‐Pincus, 2008; Zhu, Srivastava, Smith, & Martin, 2012). But even with increases in area burned or fire intensity, models also predict tree species composition shifts that pose adaptation constraints on woodpeckers (Fogg, Roberts, & Burnett, 2014) and potentially reducing habitat and biodiversity.

We propose the woodpecker guild as an ensemble of wildlife species to function as indicators of forest resiliency and biodiversity in a coupled modeled response of vegetation *and* wildlife to climate change. Woodpeckers are ideally suited as indicator species of forest ecosystem dynamics (Koch, Drever, & Martin, 2011; Segura, Castaño‐Santamaría, Laiolo, & Obeso, 2014), because they are ecologically constrained by landscape‐scale forest components, such as composition, structure, disturbance regimes, and management activities, in addition to being correlated with forest avifauna community diversity (Archaux & Bakkaus, 2007; Diaz, Armesto, Reid, Sieving, & Willson, 2005; Drever, Aitken, Norris, & Martin, 2008; Patton, 1992). Woodpeckers are also strongly associated with old‐growth/structurally complex forests (Drever et al., 2008; Hannon & Drapeau, 2005; Segura et al., 2014), which sustain greater biodiversity (Mazziotta et al., 2016) and are key habitat characteristics that modulate woodpecker population responses. These include snag density (Saab, Russell, & Dudley, 2009), tree density and diameter (Dudley, Saab, & Hollenbeck, 2012), time since last burn (Covert‐Bratland, Block, & Theimer, 2006; Hannon & Drapeau, 2005; Hobson & Schieck, 1999; Saab & Dudley, 1998; Saab, Russell, & Dudley, 2007), burn severity (Covert‐Bratland et al., 2006; Saab & Vierling, 2001; Vierling et al., 2008), and beetle outbreak (Martin, Norris, & Drever, 2006; Saab et al., 2014). Because these forest components will be impacted by climate change (Allen et al., 2010; Anderson‐Teixeira et al., 2013; Parks et al., 2016; Rocca et al., 2014; Weed et al., 2013), the change will have cascading effects on woodpecker responses, rendering them viable indicators in modeling future changes to biodiversity.

We reviewed the current and predicted trends associated with climate change impacts on woodpecker responses to identify ways to integrate woodpecker and forest ecosystem models. In addition, our intent is to provide a collective baseline of woodpecker responses to current and future climate change for integrated modeling efforts to be evaluated against. To identify ways to integrate woodpecker models, we identify inconsistencies between current (observed) and predicted responses, explore the modeling causes, and identify the models pertinent to integration that will address inconsistencies. We acknowledge there are vast syntheses possible when studying the response of woodpeckers to climate change. However, the focus of this review is to seek the information to facilitate identification of the model attributes that can best serve an integrated framework of climate–woodpecker–forest modeling. Having this framework will facilitate including other biodiversity measures (e.g., other species) in future climate modeling efforts.

## 2 METHODS AND REVIEWED LITERATURE

We conducted a systematic literature review of the observed and predicted responses to climate change of 22 North American woodpecker species. We refer to woodpecker response models as any of the following: species distribution, occupancy, abundance, and demographic models. Search terms using Google Scholar and Web of Science included “avian cavity nesters climate change,” “woodpeckers climate change,” “birds climate change,” and “birds breeding climate change.” The search spanned all literature through June 2018. We included all papers that modeled the effects of climate change on woodpecker responses. Models that based predictions on alternative analyses to evaluated datasets (Distler, Schuetz, Velásquez‐Tibatá, & Langham, 2015; Rodenhouse et al., 2008; Schuetz et al., 2015) or reported woodpecker responses aggregated at the community level (Stralberg et al., 2009) were excluded, because they did not provide individual species responses, or were redundant data.

There were a limited number of woodpecker models (studies *n* = 7; Table 1) that predicted future responses to climate change. These were mostly bioclimatic niche models (Table 1) and predicted changes to the breeding and/or winter geographic range, abundance, demographic and dispersal responses, niche temperature gradients, secondary responses inferred from range projections (species richness and niche flexibility), and species climate vulnerability (sensitivity, exposure, adaptive capacity; Supporting information Table S1). These projections all used one or more climate variables (temperature, precipitation, bioclimatic), and several included nonclimate variables (tree species occurrence, elevation, latitude, plant functional types, land use, biological traits, and survey effort; Supporting information Table S2). Because the studies used a range of climate models and/or greenhouse gas (GHG) emissions scenarios, we attempted to compare across similar GHG emissions scenarios, acknowledging the range of responses and, when possible, providing the average response.

**Table 1:** The reviewed studies of woodpecker predictions to climate change

Studies	Geographic location	Prediction period (out to)	Study season	Training/informing data source	Spatial grain of prediction	Climate models	Emissions scenarios	Number of woodpecker species	Conceptual model intersection (Figure 3)
Bancroft et al. (2016)	Fort Benning, Georgia, USA	2100	Breeding	Collected	2,500 m^2^	CCSM3, CGCM3.1, UKMO‐HadleyCM3	B1, A1B, and A2	1	D
Foden et al. (2013)	Global	NA	NA	Expert Opinion	NA	NA	NA	22	NA
Langham et al. (2015)	United States and Canada	2100	Breeding/Nonbreeding	BBS and CBC	100 km^2^	CCCMA‐CGCM3.1T47, CSIRO‐Mk3.0, IPSL‐CM4, MPI‐ECHAM5, NCAR‐CCSM3.0, UKMO‐HadleyCM3, UKMO‐HadleyGEM1, NIES	B2, A1B, and A2	22	B
Matthews et al. (2011)	Eastern United States (east of the 100th meridian)	2100	Breeding	BBS	400 km^2^	HadleyCM3, GFDL CM2.1, PCM	B1 and A1 fi	5	D
Rodenhouse et al. (2008)	Northeast United States	2100	Breeding	BBS	400 km^2^	HadleyCM3, GFDL CM2.1, PCM	B2 and A1 fi	5	B
Ralston and Kirchman (2013)	New York, Vermont, and New Hampshire, USA	2080	Breeding	ORNIS and GBIF	NR	HadleyCM3	B2 and A2	2	B
Tremblay et al. (2018)	Eastern Canada	2100	Breeding	Previous Research	250 m^2^	CanESM2	RCP 2.6, 4.5, and 8.5	1	D

Note

BBS: Breeding Bird Survey; CBC: Christmas Bird Count; Collected: data from study; GBIF: Global Biodiversity Information Facility.

Observed woodpecker responses to climate change (studies, *n* = 14; Table 2) were largely statistically based and included a variety of dependent variables to characterize a suite of woodpecker species responses in the breeding and nonbreeding seasons (Supporting information Table S3). These responses included range shifts (elevation, latitude, longitude), niche tracking, migration timing, community composition, energetic demand, and reproductive timing/performance. A few studies implicitly evaluated climate effects on avian responses via overall range shifts. Among the explicit climate effect models, the explanatory variables included climate variables (temperature, precipitation, and extremes (seasonal and annual minimums and maximums)), their aggregates (e.g., bioclimatic variables), and physiography variables (e.g., snow depth). Some studies included non‐climate explanatory variables such as habitat (land use), home range, population trends, and individual characteristics (body condition, age, breeding experience, inbreeding status, mean clutch size, diet breadth, and territory type; Supporting information Table S4).

**Table 2:** The reviewed studies of observed woodpecker responses to climate change

Study	Study period	Study season	Data source	Geographic location	Number of woodpecker species
Bateman et al. (2016)	1950–2011	Breeding	BBS	Contiguous United States	15
Hitch and Leberg (2007)	1967–1971 and 1998–2002	Breeding	BBS	BBS Central and East regions	2
Huang et al. (2017)	1969–2012	Breeding	BBS	Contiguous United States and southern Canada	7
La Sorte and Jetz (2012)	1975–2009	Nonbreeding	CBC	Between 25^◦ ^and 49^◦^ *N* latitude	4
La Sorte and Thompson III (2007)	1975–2004	Nonbreeding	CBC	Contiguous United States, Canada, and Mexico	13
La Sorte et al. (2009)	1975–2001	Nonbreeding	CBC	Contiguous United States and southern Canada	18
Prince and Zuckerberg (2015)	1989–2011	Nonbreeding	PFW	Eastern North America (below 50^◦^ *N* latitude E of the 100th meridian)	5
Schiegg et al. (2002)	1986–1998	Breeding	Collected	South‐central North Carolina, USA	1
Stephens et al. (2016)	1980–2010	Breeding	BBS	Contiguous United States	20
Tingley et al. (2009)	1911–1929 and 2003–2008	Breeding	Collected	Sierra Nevada of California	6
Tingley et al., 2012)	1911–1929 and 2006–2009	Breeding	Grinnell Resurvey Project	Sierra Nevada of California	9
Wiebe & Gerstmar, 2010)	1998–2009	Breeding	Collected	Riske Creek, British Columbia	1
Zuckerberg et al. (2009)	1980–1985 and 2000–2005	Breeding	New York State BBA	New York State	6
Zuckerberg et al. (2011)	2007–2008	Nonbreeding	PFW	Northeastern United States and adjacent Canadian provinces	4

Note

BBS: Breeding Bird Survey; BBA: Breeding Bird Atlas; CBC: Christmas Bird Count; PFW: Project Feeder Watch; Collected: data from study.

## 3 PREDICTED WOODPECKER RESPONSES TO CLIMATE CHANGE

Generally, geographic forecasts indicate a north–northeast shift of eastern U.S. avifauna species by 2100 (Matthews, Iverson, Prasad, & Peters, 2011) and a concurrent change in community composition (Langham, Schuetz, Distler, Soykan, & Wilsey, 2015; Stralberg et al., 2009). By 2080, breeding bird assemblages of northern Canada and Alaska may gain as many as 80 species, while the greatest species loss is predicted along the Canadian–U.S. border and through the Rocky Mountains (Langham et al., 2015). Model results show that the resulting dissimilarity to contemporary species composition will be greatest throughout Canada and the Rockies. These trends will downscale to regional extents; for example, upwards of 57% of California may have novel breeding bird species assemblages by 2070 with no current analogs (Stralberg et al., 2009). In addition, central and southern California are areas of peak losses of species in the nonbreeding season (Langham et al., 2015).

**Figure 1:**
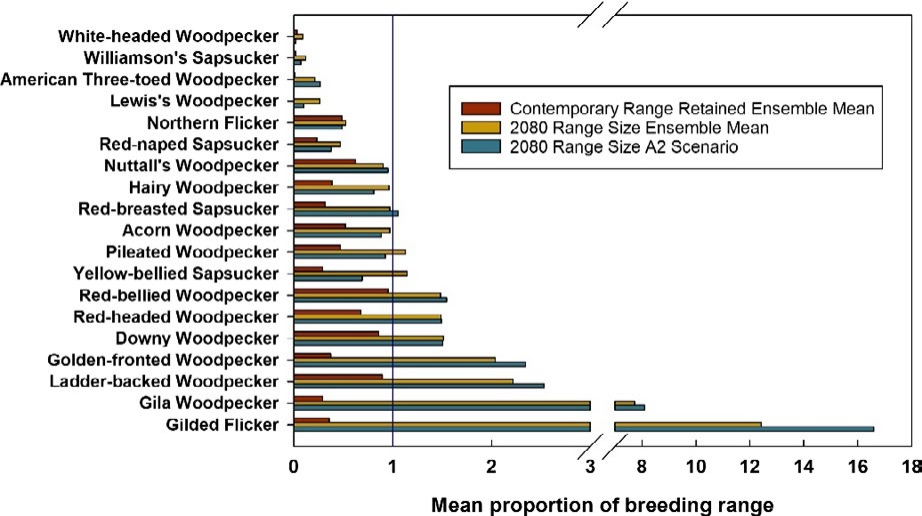
The mean proportion of North American contemporary woodpecker breeding range retained by the end of the century based on the ensemble global climate model emissions scenarios (B2, A1B, and A2: listed from low to high emissions). The overall proportional change of the breeding range by 2080 compared to 2000 based on the high emissions climate model scenario (A2) and emissions scenario ensemble means (B2, A1B, and A2). Values <1 represent a decline. Data from Langham et al. (2015)

**Figure 2:**
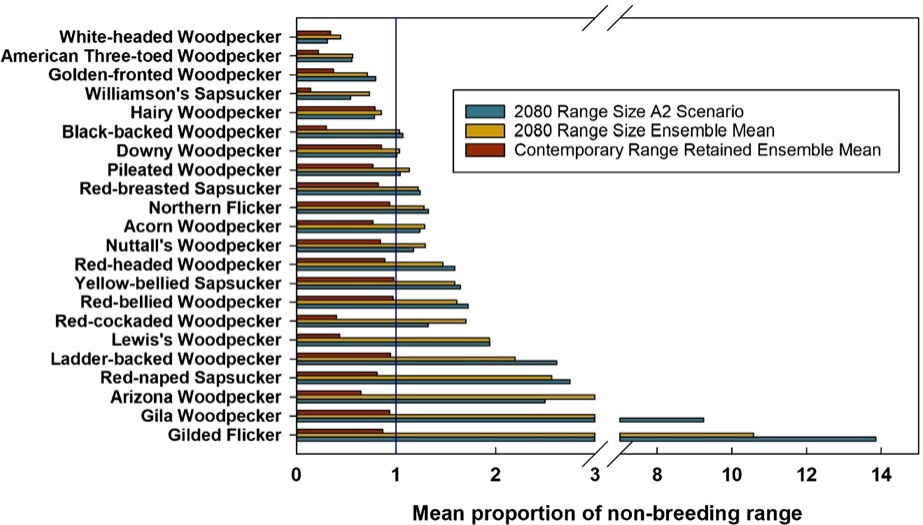
The mean proportion of North American contemporary woodpecker nonbreeding range retained by the end of the century based on the ensemble global climate model emissions scenarios (B2, A1B, and A2: listed from low to high emissions). The overall proportional change of the wintering range by 2080 compared to 2000 based on the high emissions climate model scenario (A2) and emissions scenario ensemble means (B2, A1B, and A2). Values <1 represent a decline. Data from Langham et al. (2015)

Among the models reviewed, the model of Langham et al. (2015) is the most comprehensive in relation to the greatest number of species and spatial extents modeled. The authors predict distributional changes to 2100 and compare these to species distributions in 2000 using bioclimatic modeling under a range of climate change scenarios for North American avifauna, including 20 North American woodpecker species. They used 13 combinations of emissions scenarios and general circulation models over three time periods to produce 39 different climate futures. All woodpeckers’ contemporary breeding and winter geographic ranges are predicted to contract due to climate change (Figures 1 and 2), and 13 of the 20 woodpecker species evaluated are predicted to be climate endangered or threatened due to loss of breeding and/or wintering range by the end of the century (Supporting information Table S1). Some of the range losses will be mitigated by climatically suitable range expansions. This results in an overall 53% and 23% of the woodpecker species breeding and nonbreeding ranges to exhibit net contractions by 2080, respectively (Figures 1 and 2). Overall, all woodpecker species will lose climatically suitable habitat by the end of the century, and even with net gains, a majority are labeled as climate threatened or endangered based on climatic range changes (Supporting information Table S1).

In comparison, a trait‐based assessment of climate change vulnerability via assessment of sensitivity, exposure, and adaptability found a mixed response among woodpeckers to those metrics. Most North American woodpecker species are sensitive to climate change. However, all are ranked as low vulnerability because of exposure (“the extent of the species’ environment that will change”) and/or high adaptive capacity (“the species’ ability to avoid the negative impacts of climate change through dispersal and/or micro‐evolutionary change”; Supporting information Table S1; Foden et al., 2013). This discrepancy between the bioclimatic niche predictions (Langham et al., 2015) and climate vulnerability assessments (trait‐based assessment; Foden et al., 2013) may be explained by the inclusion of measures of sensitivity and adaptability in the trait‐based evaluation. Though a qualitative assessment, the trait‐based vulnerability metric exposure to climate change (the quantified metric in bioclimatic niche models) is further modulated by a species’ sensitivity and adaptability to derive vulnerability. Bioclimatic niche models quantitatively assess the exposure of a species with minimal inclusion of the other measures of climate vulnerability (i.e., sensitivity and adaptability). Hence, a species may be exposed to shifts in climatically suitable habitat but may have adaptability potential via phenotypic plasticity or not be sensitive to the degree of climate change represented in the bioclimatic niche model.

Spatially, there is an emergent pattern of predictions among woodpeckers relative to their contemporary distributions. The climatically suitable ranges of species with contemporary northern or western distribution centroids (i.e., those associated with conifer/boreal forests) are projected to contract (Langham et al., 2015). This is in concordance with other model results of climate‐induced declines in avifauna abundance and species richness in conifer/boreal habitats of North America (Stralberg et al., 2015) and Europe (Virkkala, Heikkinen, Leikola, & Luoto, 2008). Most avian species with breeding range distributions that are associated with eastern deciduous woodlands/forests and southern mixed pine forest are predicted to be climate stable. This includes projections of the Red‐headed Woodpecker (*Melanerpes erythrocephalus*), Red‐bellied Woodpecker (*Melanerpes carolinus*), Downy Woodpecker (*Picoides pubescens*), and Pileated Woodpecker (*Hylatomus pileatus*; Langham et al., 2015; Matthews et al., 2011; Rodenhouse et al., 2008). However, species at the southern edge of their range within this region (e.g., American Three‐toed Woodpecker (*Picoides dorsalis*) and Black‐backed Woodpecker (*Picoides arcticus*)) may diminish because of the encroachment of hardwoods from lower elevations into their primary habitat (spruce‐fir; Rodenhouse et al., 2008). Nevertheless, coastal and southern regions of the United States are predicted to provide climates amenable to many wintering species (Schuetz et al., 2015).

## 4 OBSERVED WOODPECKER RESPONSES TO CLIMATE CHANGE

Generally, avian species across the globe are exhibiting behavioral and phenological shifts in response to climate change via an advancement in migration timing (Ahola et al., 2004; Hüppop & Winkel, 2006; Jenni & Kéry, 2003; Miller‐Rushing, Lloyd‐Evans, Primack, & Satzinger, 2008; Vegvari, Bokony, Barta, & Kovacs, 2010) and breeding date (Crick & Sparks, 1999; Dunn, 2004; Dunn & Møller, 2014; Visser, Holleman, & Gienapp, 2006; Winkel & Hudde, 1997). The lack of adaptation to current climate change is causing some avian population declines, possibly due to the mistiming between resource availability (e.g., prey) and migration timing (Møller, Rubolini, & Lehikoinen, 2008). Although the functional pathways of these mechanisms (i.e., phenotypic plasticity and microevolution) are not fully understood, some individuals and populations do appear to be responding to climate change, and phenotypic plasticity appears to mitigate fitness loss due to these changes (Gienapp, Teplitsky, Alho, Mills, & Merilä, 2008).

Laying date advancement and increase in reproductive productivity of Northern Flickers (*Colaptes auratus*) were observed along the U.S. Pacific coast (Wiebe & Gerstmar, 2010). The authors showed that the response is spatially explicit; it correlates with increases in local ambient temperatures instead of broad regional climate indices or range‐wide temperature gradients. Moreover, differing climatic conditions is producing similar phenology responses within the same species. Red‐cockaded Woodpeckers (*Leuconotopicus borealis*) are laying earlier, and those that do are more productive (Schiegg, Pasinelli, Walters, & Daniels, 2002). The climate factors that correlate to these responses differ between populations; one population is responding to increases in temperature and the other increases in precipitation (Schiegg et al., 2002). Mechanistically, this may be occurring via genetic diversity and age‐based experience, which increases plasticity (Schiegg et al., 2002). Woodpecker phenology may be shifting in response to changing climatic conditions; however, behavioral plasticity may not always mitigate climate vulnerability.

Climate change effects manifested via habitat suitability change are not producing behavioral plasticity responses among some woodpeckers. In the southwest United States, lack of behavioral plasticity caused Northern Flicker, Red‐naped Sapsucker (*Sphyrapicus nuchalis*), Williamson's Sapsucker (*Sphyrapicus thyroideus*), Hairy Woodpecker (*Leuconotopicus villosus*), Downy Woodpecker, and Acorn Woodpecker (*Melanerpes formicivorus*) populations to decline significantly, correlating with the climate change‐induced density decline of quaking aspen (*Populus tremuloides*; Di Orio, Callas, & Schaefer, 2005; Worrall et al., 2008, 2013), their preferred nesting tree (Martin, 2015). This is rendering some species more vulnerable because of sensitivity to changes in nesting tree availability and lack of observed adaptability. Martin (2015) noted that resource specialization and scale‐dependent habitat selection will be important factors in species population responses to climate‐induced habitat change. This means that accounting for such ecological niche shifts (i.e., loss of nesting trees) and subsequent habitat selection in models is important to capture the vulnerability of species and biodiversity dynamics of an ecosystem.

In response to changing climatic conditions, avifauna geographic breeding (Chen, Hill, Ohlemüller, Roy, & Thomas, 2011; Hitch & Leberg, 2007; Hovick et al., 2016; Matthews, O'Connor, Iverson, & Prasad, 2004; Parmesan & Yohe, 2003; Thomas & Lennon, 1999; Tingley et al., 2012) and nonbreeding (La Sorte & Jetz, 2012; La Sorte & Thompson III, 2007) distributions are shifting. Though most woodpecker populations are increasing, distribution shifts in relation to ongoing climate change are heterogeneous and differ across spatial and temporal scales (Supporting information Table S3; Bateman et al., 2016; Hitch & Leberg, 2007; Huang, Sauer, & Dubayah, 2017; La Sorte & Thompson III, 2007; Tingley et al., 2012; Tingley, Monahan, Beissinger, & Moritz, 2009; Zuckerberg, Woods, & Porter, 2009). Among the North American woodpecker species, these heterogeneous shifts are likely confounded by abundance changes, because based on Breeding Bird Survey and Christmas Bird Count data, most woodpecker populations have been increasing in the last four decades (Supporting information Figures S1 and S2; Sauer et al., 2017; Soykan et al., 2016).

Studies that have specifically evaluated woodpeckers (*n* = 8) have found geographic and elevational shifts (Supporting information Table S3), and most woodpecker range extents are either expanding or not changing with the exception of the contracting Ladder‐backed Woodpecker (*Dryobates scalaris*), Williamson's Sapsucker, and Red‐headed Woodpecker (Bateman et al., 2016). Stephens et al. (2016) found that 13 of the 20 woodpecker species included in their comprehensive avifauna study have been advantaged by climate change across most of the evaluated states; that is, the probability of occurrence was positively associated with climatic trends and was independent of abundance trends (Supporting information Table S3). It has been hypothesized that as yearly mean temperatures rise, breeding and nonbreeding ranges in North America will likely continue to track climatically suitable habitat north and only be constrained by terrestrial habitat features (La Sorte & Jetz, 2010). Though over the last four decades, avifauna have not always tracked their climatic niches; there has been a lag effect in some North American species (La Sorte & Jetz, 2012). In some instances, species that have colonized human‐dominated systems do not fully track their climatic niche shifts (Tingley et al., 2009).

The complexity of woodpecker range responses can be appreciated by comparing several species. Only the Red‐headed Woodpecker (decreased distribution at southern range edge) and Red‐bellied Woodpecker (expansion at northern range edge and northwest range centroid shift) had the same directional response among the breeding and nonbreeding seasons, respectively (Supporting information Table S3; Bateman et al., 2016; Huang et al., 2017; La Sorte & Thompson III, 2007; Zuckerberg et al., 2009). The distribution contraction of the Red‐headed Woodpecker and expansion of the Red‐bellied Woodpecker are consistent with them being climate disadvantaged and advantaged, respectively (Supporting information Table S3; Stephens et al., 2016). In contrast, the Yellow‐bellied Sapsucker (*Sphyrapicus varius*) shifted south (Hitch & Leberg, 2007; Zuckerberg et al., 2009) and east during the breeding season (Bateman et al., 2016), but tracked the mean winter temperature increases northward during the nonbreeding season (La Sorte & Thompson III, 2007). The increase in Yellow‐bellied Sapsucker breeding season abundance between 2005 and 2015 within the United States (Sauer et al., 2017) is concurrent with a southern and eastern range shift but appears independent of climatic shifts. Based on the breeding distribution of the Yellow‐bellied Sapsucker between 1980 to 2010 and independent of abundance trends, it is considered disadvantaged by climate change in a majority of the states evaluated (Supporting information Table S3; Stephens et al., 2016). In addition, the northward winter range shift is occurring without a concurrent population abundance change (Supporting information Figure S1; Soykan et al., 2016). The Yellow‐bellied Sapsucker, in contrast to Red‐headed Woodpecker and Red‐bellied Woodpecker range changes explained by climate, highlights the complexity of climate‐based range changes; climate is expected to increase the vulnerability of this species even though it is not inducing observed range and population dynamics.

Generally, North American winter avifauna species richness and the average body mass of community assemblages are increasing (Supporting information Table S3; La Sorte, Lee, Wilman, & Jetz, 2009). In eastern North America, winter bird occupancy is being climatically constrained (Zuckerberg et al., 2011) and community assemblages are becoming dominated by warm‐adapted species as mean winter temperature increases (Prince & Zuckerberg, 2015). The northward winter range shift of the Pileated Woodpecker, Red‐bellied Woodpecker, Northern Flicker (larger bodied woodpeckers), and Yellow‐bellied Sapsucker is strongly contributing to these winter community composition changes (Prince & Zuckerberg, 2015). However, only the Pileated and Red‐bellied Woodpecker populations, both resident migrants, exhibited a concurrent increase in abundance during the winter season (Supporting information Figure S1; Soykan et al., 2016). In the context of modeling, associated climate change‐induced community‐scale dynamics over time are not necessarily in agreement with spatial climatic trends; that is, under the auspice of climate change, observed spatial gradients relating to climate may not accurately predict temporal trends of species assemblages at the community scale (La Sorte et al., 2009).

Montane environments of the western United States are losing breeding season avifauna diversity at all elevational gradients (Tingley & Beissinger, 2013), and latitude and elevation range shifts have been idiosyncratic (Auer & King, 2014). Among the studies reporting elevation climate space tracking (Tingley et al., 2012; Zuckerberg et al., 2009), woodpeckers responded heterogeneously (Supporting information Table S3). In the Sierra Nevada of California, avifauna with low and high elevation range centroids tend to track favorable precipitation and temperature conditions (Tingley et al., 2012, 2009) shifting species upslope and downslope, respectively (Tingley et al., 2012). Comparing 1911–1929 to 2003–2009, Tingley and Beissinger (2013) found avian populations decreased across all elevational gradients, species richness was lower, and compositions changed. However, woodpecker responses differed slightly from the community response with more than half not declining. The adaptive capacity of these woodpeckers is considered high (Supporting information Table S1; Foden et al., 2013), so climate change alone may not drive responses and community dynamics may not scale to the species level. Thus, accounting for two‐dimensional climate space interactions (Tingley et al., 2012) and subsequent niche constraints in models is important for montane populations.

The described range shifts and behavioral responses likely reflect complex interactions between climate, habitat changes, and anthropogenic influences (La Sorte & Thompson III, 2007) that will affect future population dynamics. For example, the Red‐bellied Woodpecker's range expansion north between 1966 and 2009 (Bled, Sauer, Pardieck, Doherty, & Royle, 2013) was attributed to maturing forest, backyard bird feeders, (Jackson and Davis Jr 1998; Meade, 1988), and planted trees in the Great Plains (Shackelford, Brown, & Conner, 2000). Although climate is likely influencing these broad‐scale range changes and expansions, it is difficult to ascribe change to climate, if it can be explained by other spatially explicit variables, for example, habitat patterns (Bled et al., 2013). Currie and Venne (2017) found that among some passerines, their realized niche temperatures have changed in the last three decades and that represents changes in ambient temperature and not necessarily species movements. That is, species did not maintain more constant thermal niches through time or exhibit strong poleward shifts especially at the higher latitudes; therefore, climate change, more specifically temperature, is not always the major driver of continental species’ range shifts (Currie & Venne, 2017). Moreover, observed lag responses to contemporary climate change are likely to occur in the future resulting in miss‐estimations of range change based on climate condition‐only models (Hovick et al., 2016; La Sorte & Jetz, 2012; La Sorte et al., 2009). Factors other than broad‐scale climate are confounding distribution and habitat use responses. The mechanisms underlying observed shifts are numerous (Currie & Venne, 2017; Hitch & Leberg, 2007; Hovick et al., 2016; La Sorte & Thompson III, 2007; Tingley et al., 2009) and require further consideration, especially within modeling frameworks, if climate‐induced distribution changes are to be accurately predicted.

## 5 COMPARING CLIMATE‐INDUCED OBSERVED AND PREDICTED TRENDS

We found that 7 of 15 species short‐term breeding geographic range predictions under one or both emissions scenarios are not in agreement with observed trends (Table 3). The contemporary breeding ranges of the Williamson's Sapsucker, Ladder‐backed Woodpecker, and Red‐headed Woodpecker are contracting, and the Golden‐fronted Woodpecker (*Melenerpes aurifrons*), Lewis's Woodpecker, Red‐breasted Sapsucker, and White‐headed Woodpecker (*Picoides albolarvatus*) ranges are stable. In addition, the American Three‐toed Woodpecker climatically suitable range is predicted to contract substantially in the short term (Table 3); however, observed trends from 2005 to 2015 indicate an increasing population (Sauer et al., 2017). The disagreements between short‐term predictions and observed trends highlight the potential incongruencies between future potential climatic niches and realized niches based on climate–woodpecker bioclimatic niche models.

**Table 3:** The predicted 2020 breeding range size relative to the 2000 range (Langham et al., 2015) and observed contemporary breeding range changes (Bateman et al., 2016)

Species	Predicted breeding		Observed breeding
	High emissions	Low emissions	
Acorn Woodpecker	1.37	1.25	Expanding
American Three‐toed Woodpecker	0.30	0.27	NA
Arizona Woodpecker	NA	NA	NA
Black‐backed Woodpecker	NA	NA	NA
Downy Woodpecker	1.15	1.18	Expanding
Gila Woodpecker	3.29	3.64	Expanding
Gilded Flicker	3.12	2.83	NA
Golden‐fronted Woodpecker	0.71*	0.95	No change
Hairy Woodpecker	0.92	0.97	No change
Ladder‐backed Woodpecker	1.49*	1.56*	Contracting
Lewis's Woodpecker	0.84*	0.89*	No change
Northern Flicker	0.96	0.83	NA
Nuttall's Woodpecker	0.97	0.93	No change
Pileated Woodpecker	1.25	1.27	Expanding
Red‐bellied Woodpecker	1.15	1.15	Expanding
Red‐breasted Sapsucker	0.95	0.82*	No change
Red‐cockaded Woodpecker	NA	NA	NA
Red‐headed Woodpecker	1.07*	1.08*	Contracting
Red‐naped Sapsucker	1.08	0.83	NA
White‐headed Woodpecker	0.73*	0.67*	No change
Williamson's Sapsucker	1.55*	0.92*	Contracting
Yellow‐bellied Sapsucker	1.44	1.62	Expanding

Notes

Breeding predictions that disagree (>10% difference from 1) are noted with *. Emissions scenarios are the A2 (high) and B2 (low) IPCC SRES.

We hypothesize that woodpecker responses derived from climate–woodpecker models are likely not in agreement with observed trends because additional niche characteristics (e.g., forest composition) are responding differently to climate change, and these changes are not represented in the models being used. Therefore, mismatches in observed and future trajectories will continue to arise as actual vegetation cover (i.e., habitat) differs from theoretical because of climate conditions interacting with landscape‐scale processes (e.g., fire, seed dispersal; Hampe & Jump, 2011). A comparison between climate–woodpecker model projections and habitat responses of such species in climate–forest models emphasizes the potential for such inconsistencies.

For example, western montane and boreal woodpecker species such as the American Three‐toed Woodpecker, Red‐naped Sapsucker, Williamson's Sapsucker, and White‐headed Woodpecker are predicted to lose climatically suitable habitat based on the bioclimatic niche models (Figures 1 and 2; Supporting information Table S1). Climate–forest models associated with these woodpeckers’ habitats project shifts in species distribution and composition (McKenney, Pedlar, Lawrance, Campbell, & Hutchinson, 2007). In other words, climate–woodpecker models indicate a range loss due to climate change, but climate–forest models report a mixed response of the underlying habitat. Assuming tree species of this region (associated with woodpeckers’ suitable habitat) track their climate niches (i.e., the climatically suitable range of woodpeckers is more closely associated with a congruent shift in vegetation), forest composition change projections are mixed leading to the potential for habitat persistence. Lodgepole pine (*Pinus contorta*), black spruce (*Picea mariana*), and aspen geographic ranges will likely decline (Coops & Waring, 2011a, 2011b; McKenney et al., 2007; Rehfeldt, Ferguson, & Crookston, 2009), ponderosa pine *(Pinus ponderosa)* range projections show mixed results (Coops & Waring, 2011b; McKenney et al., 2007), and Douglas fir (*Pseudotsuga menziesii*) range is predicted to increase (Coops & Waring, 2011b; McKenney et al., 2007). However, tree species will exhibit some level of delayed climate niche tracking (McKenney et al., 2007) because tree species migration will likely not keep pace with projected climate change (L. R. Iverson, Schwartz, & Prasad, 2004). This will result in a lag effect between changing climatically suitable geographic range and subsequent woodpecker species colonization because contemporary vegetation patterns will not perfectly track climatic shifts. This will increase the likelihood of the persistence of suitable habitat or refugia (Beever et al., 2016) through the 21st century, which are undetectable with bioclimatic niche models (Wiens & Bachelet, 2010).

Using climatic conditions associated with contemporary distributions can under‐predict the areas that are suitably post‐climatic change (Early & Sax, 2014) because landscape‐scale processes can cause a lag in vegetation (Wu et al., 2015) or animal (Menéndez et al., 2006) responses. Processes that create a mismatch between expected and actual vegetation could result in the persistence of suitable habitat patches that mitigate short‐term climate change pressures on some populations (Kellermann & van Riper, 2015). For example, fire potential and frequency are predicted to increase across most of the United States and more specifically the Rocky Mountains (Liu, Goodrick, & Stanturf, 2013; Rocca et al., 2014). This is proposed to fundamentally change the western U.S. fire regime to dynamics not observed in the historical and paleoecological record, that is, a novel fire–climate–vegetation relationship is predicted (Westerling, Turner, Smithwick, Romme, & Ryan, 2011). Bioclimatic range projections can track climate change assuming processes occurring under current climatic conditions persist. However, bioclimatic niche models do not fully capture the shifting woodpecker niche constraints resulting from novel climate‐vegetation‐disturbance interactions. It is possible that increases in fire severity and or frequency may be beneficial to some woodpecker species in the western United States (Hutto & Patterson, 2016) and that climatic changes that do not pose direct physiological constraints on woodpeckers may result in suitable habitat via forest composition and structure changes. Therefore, accounting for vegetation and the ecosystem processes underlying vegetation dynamics is important in the climate–woodpecker–forest integration framework.

There are instances where climate–woodpecker models agree with observed trends, and future predictions are supported by climate–forest projections of the underlying habitat vegetation composition. However, the mechanisms underlying these observed and predicted trends are nuanced and identifying them will improve model integration. For example, the Yellow‐bellied Sapsucker has short‐term predictions that are in agreement with observed trends (Table 3) and long‐term predictions indicate range contractions (Langham et al., 2015; Matthews et al., 2011). The Yellow‐bellied Sapsucker has been increasing in abundance at its southern range extent since 1966 (Sauer et al., 2017), shifting south, expanding east, and increasing in geographic range (Bateman et al., 2016; Hitch & Leberg, 2007; Zuckerberg et al., 2009), though this is despite climatic factors (Supporting information Table S3; Stephens et al., 2016). They favor early‐successional forests and are currently increasing because of the reversion of post‐European settlement agricultural land use to forests (Walters, Miller, & Lowther, 2002). The contemporary geographic breeding range is projected to decrease by 2080 and shift north under the highest emissions scenario (A2 model; Figure 1); this will result in an overall geographic range reduction of 31% (Langham et al., 2015) and a breeding range almost entirely in Canada (National Audubon Society, 2017). Further, the predicted decline (Supporting information Table S1) is in agreement with results from a climate–woodpecker–forest model for the eastern and northeastern regions of the United States (Matthews et al., 2011; Rodenhouse et al., 2008), which represents the southern portion of the breeding range.

This predicted decline of the Yellow‐bellied Sapsucker climatically suitable range appears to be supported by climate–forest projections. The tree species most associated with their mixed‐forest breeding habitat (quaking aspen (*Populus tremuloides*), red maple (*Acer rubrum*), yellow birch (*Betula alleghaniensis*), and paper birch (*Betula papyrifera*); Walters et al., 2002) will shift north with concurrent contractions in climatically suitable ranges (except: red maple range will increase) according to bioclimatic niche tree models (McKenney et al., 2007). Southern limited species (e.g., sugar maple (*Acer saccharum*), American basswood (*Tillia americana*), and bitternut hickory (*Carya cordiformis*); McKenney et al., 2007; Terrier, Girardin, Perie, Legendre, & Bergeron, 2013) will expand north, causing a tree composition change toward more deciduous dominance (Terrier et al., 2013).

Although these climate–forest bioclimatic niche tree models may suffer from under‐prediction errors (Early & Sax, 2014), a process‐based model of these forest ecosystems indicates a seral stage shift (Thompson, Foster, Scheller, & Kittredge, 2011), which will affect Yellow‐bellied Sapsucker habitat suitability. The contemporary early‐successional forests of the northeast United States will change by midcentury; at the southern edge of the Sapsucker's breeding range, a shift toward late‐successional species is expected and possibly accelerated as climate change has a net positive impact on growth (Thompson et al., 2011). In addition, the contemporary Sapsucker population is likely above historical size because of the large‐scale changes in land use post‐European colonization (Walters et al., 2002). It is likely the current population size and range extents are not sustainable because of antecedent land use change and forest succession; however, climate change will synergistically interact with successional trajectories.

The predicted declines of climatically suitable range of the Yellow‐bellied Sapsucker appear to be consistent with shifts in climate‐induced tree species composition and forest successional dynamics. Although short‐term climate–woodpecker predictions agree with observed trends, climate is not underlying this trend. Thus, climate–woodpecker predictions may not fully capture future dynamics. Contemporary range distributions are likely a function of forest vegetation shifts, due to historic land use. Future distributions will likely be a function of vegetation shifts resulting from climate change interactions with forest succession. Capturing the effects of climate and forest successional dynamics in the integrated framework of climate–woodpeckers–forest modeling will help account for more nuanced distribution responses.

As the niche constraints (e.g., forest composition, structure) associated with woodpeckers respond to climate change (Ganey & Vojta, 2012; Westerling, Hidalgo, Cayan, & Swetnam, 2006), climate variables may poorly approximate woodpecker species responses compared to measures of ecosystem dynamics, for example, forest net primary productivity (Tingley et al., 2009) or forest composition. Therefore, ecosystems predicted to be climatically unsuitable (per bioclimatic niche models) but predicted to maintain or increase key habitat species or functions (per process‐based climate–forest models) may still be suitable habitat for woodpeckers because of resource persistence. Accounting for associated niche constraints in a climate–woodpecker–forest modeling framework will produce more informative responses.

## 6 FRAMEWORK INTEGRATION

**Figure 3:**
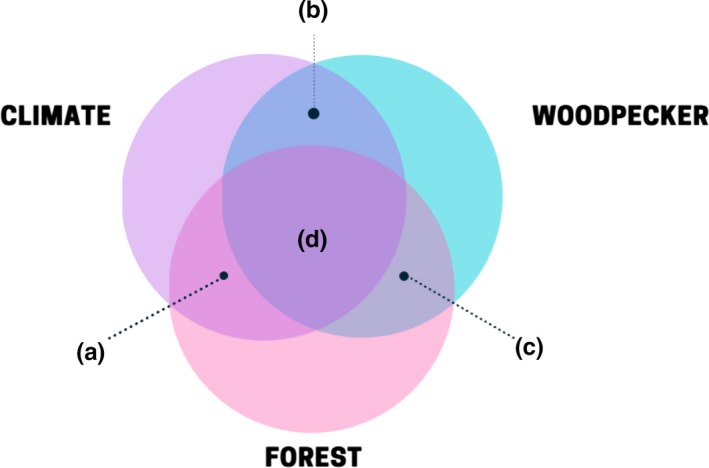
The integrated framework of climate–woodpecker–forest modeling (d) resulting from the linking of separate model types (a–c). (a) Climate–forest prediction models include a spectrum of model types: dynamic global vegetation models (DGVMs) to GAP models to dynamic community process‐based forest landscape models (i.e., dynamic communities, spatial interactions, and ecosystem processes); (b) Climate–woodpecker prediction models include bioclimatic envelope models; (c) Woodpecker–forest models include realized niche models (e.g., occupancy), potential niche models (e.g., habitat suitability), and demographic models

Development of forest management strategies aimed at increasing or preserving wildlife species in a changing climate requires modeling efforts that include the coupled response of vegetation *and* wildlife to climate change. We suggested woodpeckers as indicator species of forest resiliency and biodiversity in an integrated forest–wildlife modeling framework, because they are ecologically constrained by forest structure, composition, and processes, which also affect a diversity of other organisms. Based on our comparison of predicted and observed woodpecker responses to climate change, we propose a framework for integration of climate, woodpecker, and forest modeling (Figure 3).

Models used to project future abundances and distributions of North American woodpecker species have largely been developed independently of process‐based models of forest vegetation responses to climate change (Table 1; Figure 3). The available bioclimatic niche models that predominate the predictions about woodpeckers (Figure 3b) provide potential broad‐scale range distribution trends (Pearson & Dawson, 2003); however, they lack the finer scale habitat details (e.g., forest structure, composition, and habitat characteristics) that affect localized woodpecker population responses and may strongly interact with climate change. Habitat use and population persistence in a changing climate are difficult to ascertain without vegetation responses. For example, the inclusion of vegetation indices in distribution forecasts of boreal and mixed conifer forests avifauna is important for improving modeling results (Cumming et al., 2014). The complexities of climate, vegetation, and disturbance interactions that modulate woodpecker habitat use underscore the need for coupled modeling that accounts for these ecological details (La Sorte & Jetz, 2010).

While the inclusion of vegetation (dynamic global vegetation model: DGVM [Figure 3a]; for a review of the spectrum of climate–forest models, see: Scheller & Mladenoff, 2007) has improved avian distribution models (Conlisk, Syphard, Franklin, & Regan, 2015; Matthews et al., 2011), plant functional types (outputs of DGVMs) still do not adequately account for future habitat distributions of woodpeckers (i.e., the type of climate–forest model (Figure 3a) is important). This is because plant functional groupings may be of a scale too course to model woodpecker responses to forest characteristics. For example, Bancroft, Lawler, and Schumaker (2016) found no impact of climate change on Red‐cockaded Woodpecker habitat loss. They modeled climate as direct (i.e., precipitation effects on reproduction) and indirect (i.e., plant functional group responses to temperature and precipitation) effect. However, the resilience of the Red‐cockaded Woodpecker population is related to the structural components of a stand (tree density and size class distributions) and ground cover composition (James, Hess, Kicklighter, & Thum, 2011), which are indistinguishable at the scale of plant functional groups. Therefore, even with the persistence of the needle‐leaved evergreen biome or long‐leaf pine successional stages within this region (Costanza, Terando, McKerrow, & Collazo, 2015), finer scale niche attributes are important (Schiegg et al., 2002) and should be included in model integration.

Dynamic community process‐based forest landscape models (Scheller & Mladenoff, 2007) such as the LANDIS models (LANDIS‐II and LANDIS PRO; Figure 3a) that incorporate finer scale climate–vegetation–disturbance interactions compared to bioclimatic DGVMs are ideally suited for this integration (Di Febbraro et al., 2015; Iverson, Prasad, Matthews, & Peters, 2011; LeBrun et al., 2016; Tremblay, Boulanger, Cyr, Taylor, & Price, 2018). These models could improve woodpecker distribution modeling, especially within the context of multi‐objective management scenarios (Martin, Hurteau, Hungate, Koch, & North, 2014). Many of the key habitat characteristics and processes (e.g., forest composition and structure, disturbance type, intensity, and temporal trends) that modulate woodpecker population responses are already output variables of forest landscape models, allowing for points of integration between the two modeling disciplines (Figure 3a,c). In addition, these models can be modulated by climate data, which is the crucial integration element in the climate–woodpecker–forest framework (Figure 3d). Integration examples support this proposed framework. LANDIS‐II model projections by Martin et al. (2014) found that managing long‐leaf pine habitat for carbon storage decreases biodiversity and Red‐cockaded Woodpecker habitat at the expense of increased carbon sequestration. Similarly, the Black‐backed Woodpecker in boreal forest of Canada are predicted to decline under climate change or business as usual harvest practices (Tremblay et al., 2018). The LANDIS models (Figure 3a) allow for climate data integration, simulate ecosystem processes that produce emergent vegetation dynamics that constrain woodpecker distributions, and output variables that can inform woodpecker–forest models (Figure 3c).

In summary, after evaluating the predicted and observed woodpecker trends associated with climate change, we found there are inconsistencies between climate–woodpecker predictions and observed woodpecker responses, highlighting the uncertainty of future woodpecker distribution and population predicted responses. We conclude that implementation of climate smart management strategies aimed at increasing or preserving wildlife species will require modeling efforts to include the coupled response of climate–wildlife–forest (Figure 3). The use of an indicator species of climate effects on forest biodiversity and resiliency is an improvement to ecosystem modeling. The general principle of coupled modeling frameworks is not a new proposal with regard to climate change (Root & Schneider, 1993). However, to date, we are aware of no model (Figure 3d) that has managed to fully combine wildlife niche modeling into a climate–forest model; meaning modeling activities have utilized multiple models in tandem with data handoffs rather than have the models interact with feedbacks to processes. Our review suggests that fully integrating climate–woodpecker–forest models will address the limitations of climate–woodpecker models, while providing a biodiversity measure for climate–forest modeling efforts. Selection of the proper models within the framework will improve the resolution of fine‐scale woodpecker population responses to climate change and support multi‐objective management through integration of a habitat evaluation metric.

## CONFLICT OF INTEREST

None declared.

## AUTHOR CONTRIBUTIONS

EW performed all data acquisition and synthesis. EW and TH wrote the manuscript. All authors contributed to editing and revising the manuscript. All authors read and approved the final manuscript.

## DATA ACCESSIBILITY STATEMENT

All summarized data in this study are available in Supplemental Tables S1‐S4.
