# Supplementary figures and images for: Climate change, woodpeckers, and forests: Current trends and future modeling needs
Source: Ecol Evol. 2019 Feb 5;9(4):2305–19. doi: 10.1002/ece3.4876 (PMC6392386; doi:10.1002/ece3.4876)

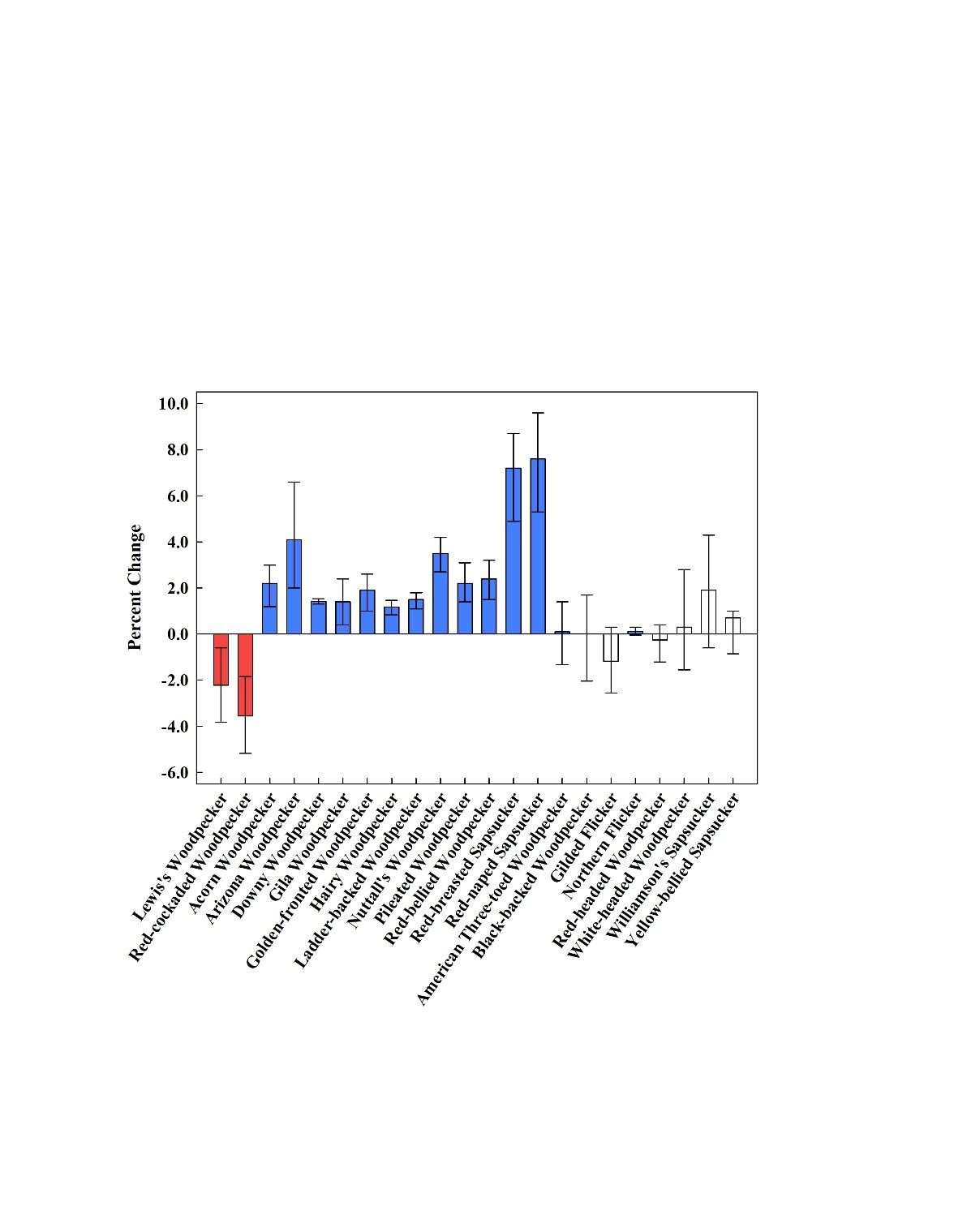

Supplement: Supplementary file 1 [file ECE3-9-2305-s001.tif]

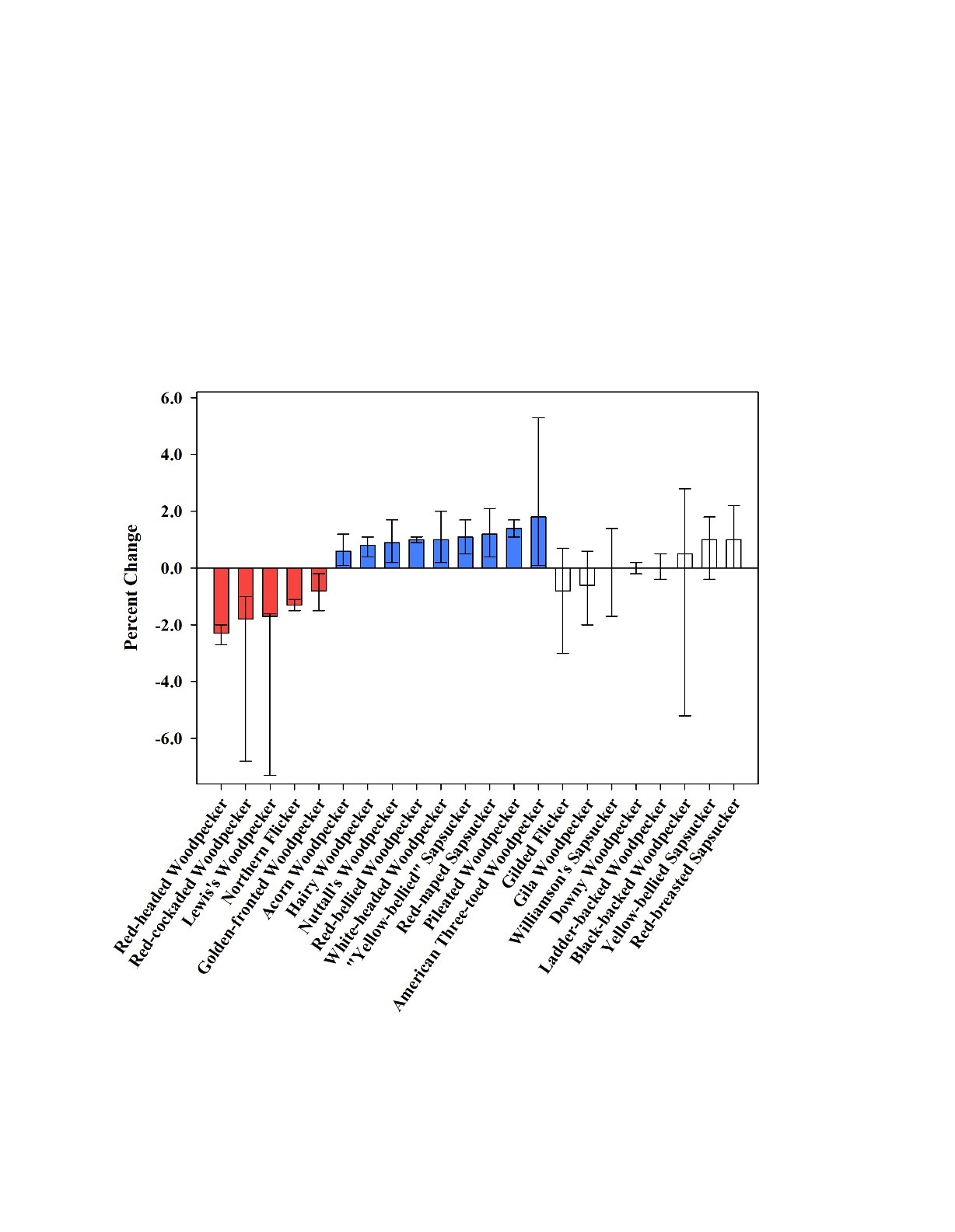

Supplement: Supplementary file 2 [file ECE3-9-2305-s002.tif]
