# Supplementary material for: Climate change, woodpeckers, and forests: Current trends and future modeling needs
Source: Ecol Evol. 2019 Feb 5;9(4):2305–19. doi: 10.1002/ece3.4876 (PMC6392386; doi:10.1002/ece3.4876)
Supplement: Supplementary file 3 [file ECE3-9-2305-s003.docx]

| **Species** | **Hi Emissions** | **Low Emissions** | **Hi Emissions** | **Low Emissions** | **Hi Emissions** | **Low** | **High Emissions Low** | | **High** | **Low** | **Breeding** | **High** | **Low** | **Hi Emissions** | **Low Emissions** | **High Emissions** | **Low Emissions** | **High Emissions** | **Low Emissions** | **Audubon** | **Change in** | **Change in** | **Change in** | **Sensitivity** | **Unadaptability** | **Exposure** | **Overall Vulnerability** |
| --- | --- | --- | --- | --- | --- | --- | --- | --- | --- | --- | --- | --- | --- | --- | --- | --- | --- | --- | --- | --- | --- | --- | --- | --- | --- | --- | --- |
|  | **(GCM Avg)** | **(GCM Avg)** | **(GCM Avg)** | **(GCM Avg)** | **(GCM Avg)** | **Emissions** | **% Breeding** | **Emissions %** | **Emissions** | **Emissions** | **Habitat** | **Emissions** | **Emissions** | **(Wintering** | **(Wintering** | **(Breeding** | **(Breeding** | **(Wintering** | **(Wintering** | **Climate** | **Productivity** | **Productivity** | **Productivity** |  |  |  |  |
|  | **(Breeding** | **(Breeding** | **(Breeding** | **(Breeding** | **(Breeding** | **(GCM Avg)** | **Area Change** | **Breeding** | **Breeding** | **Breeding** | **Climate** | **(Breeding** | **(Breeding** | **Distribution** | **Distribution** | **Distribution** | **Distribution** | **Distribution)** | **Distribution** | **Sensitivity** | **relative to year relative to year relative to year** | | |  |  |  |  |
|  | **Abundance** | **Abundance %** | **Abundance %** | **Abundance %** | **Distribution)** | **(Breeding** |  | **Area Change** | **Centroid** | **Centroid** | **Response** | **Distribution** | **Distribution** | **Change by** | **Change by** | **Change by** | **change by** | **Change by** | **change by** | **Rank** | **2000 RCP 2.6** | **2000 RCP 4.5** | **2000 RCP 8.5** |  |  |  |  |
|  | **% change)** | **change)** | **change)** | **change)** | **% Change** | **Distribution %** |  |  | **Shift (km)** | **Shift (km)** |  | **Change by** | **Change by** | **2020 relative** | **2020 relative** | **2080 relative** | **2080 relative** | **2080 relative** | **2080 relative** |  |  |  |  |  |  |  |  |
|  |  |  |  |  |  | **change)** |  |  |  |  |  | **2020 relative** | **2020 relative** | **to 2000)** | **to 2000)** | **to 2000)** | **to 2000)** | **to 2000)** | **to 2000)** |  |  |  |  |  |  |  |  |
|  |  |  |  |  |  |  |  |  |  |  |  | **to 2000)** | **to 2000)** |  |  |  |  |  |  |  |  |  |  |  |  |  |  |
| **Acorn Woodpecker** |  |  |  |  |  |  |  |  |  |  |  | **36.6%** | **25.2%** | **29.8%** | **26.1%** | **-11.5%** | **-5.2%** | **23.7%** | **13.3%** | **STABLE** |  |  |  | **H** | **L** | **L** | **L** |
| **American Three-toed Woodpecker** |  |  |  |  |  |  | **46.8** | **29.6** | **909.8** | **532** |  | **-70.1%** | **-72.8%** | **-40.9%** | **-43.8%** | **-73.3%** | **-79.3%** | **-44.3%** | **-52.7%** | **ENDANGERED** |  |  |  | **H** | **U** | **L** | **L** |
| **Arizona Woodpecker** |  |  |  |  |  |  |  |  |  |  |  | **NA** | **NA** | **295.9%** | **242.1%** | **NA** | **NA** | **149.7%** | **173.8%** | **STABLE** |  |  |  | **H** | **L** | **H** | **L** |
| **Balck-backed Woodpecker** |  |  |  |  |  |  | **18** | **25.2** | **931.2** | **1160** |  | **NA** | **NA** | **0.9%** | **9.4%** | **NA** | **NA** | **6.7%** | **-2.9%** | **THREATENED** | **81%** | **89%** | **92%** | **H** | **L** | **L** | **L** |
| **Downy woodpecker** | **-11.4** | **-11.4** | **3** | **6.6** | **0** | **0** |  |  |  |  |  | **14.8%** | **17.7%** | **-1.9%** | **-0.5%** | **50.7%** | **58.3%** | **1.0%** | **4.0%** | **STABLE** |  |  |  | **H** | **L** | **L** | **L** |
| **Gila Woodpecker** |  |  |  |  |  |  |  |  |  |  |  | **228.6%** | **263.7%** | **200.6%** | **175.9%** | **708.9%** | **595.0%** | **824.6%** | **453.7%** | **THREATENED** |  |  |  | **H** | **L** | **H** | **L** |
| **Gilded Flicker** |  |  |  |  |  |  |  |  |  |  |  | **212.4%** | **183.4%** | **239.5%** | **251.9%** | **1559.5%** | **918.3%** | **1285.8%** | **931.2%** | **THREATENED** |  |  |  | **H** | **L** | **H** | **L** |
| **Golden-fronted Woodpecker** |  |  |  |  |  |  |  |  |  |  |  | **-29.2%** | **-5.1%** | **-25.5%** | **-27.1%** | **134.5%** | **77.9%** | **-20.4%** | **-53.0%** | **THREATENED** |  |  |  | **L** | **L** | **H** | **L** |
| **Hairy Woodpecker** |  |  |  |  |  |  |  |  |  |  |  | **-7.6%** | **-3.4%** | **-6.9%** | **-4.9%** | **-18.9%** | **8.0%** | **-21.7%** | **-11.6%** | **THREATENED** |  |  |  | **H** | **L** | **H** | **L** |
| **Ladder-backed Woodpecker** |  |  |  |  |  |  |  |  |  |  |  | **49.2%** | **55.8%** | **32.9%** | **40.5%** | **153.2%** | **84.1%** | **161.5%** | **96.9%** | **STABLE** |  |  |  | **H** | **L** | **H** | **L** |
| **Lewis's Woodpecker** |  |  |  |  |  |  |  |  |  |  |  | **-15.8%** | **-10.8%** | **56.7%** | **56.7%** | **-89.9%** | **-70.1%** | **94.3%** | **70.8%** | **THREATENED** |  |  |  | **H** | **L** | **H** | **L** |
| **Northern Flicker** |  |  |  |  |  |  |  |  |  |  |  | **-4.5%** | **-16.7%** | **7.5%** | **7.5%** | **-51.2%** | **-51.8%** | **32.9%** | **26.6%** | **STABLE** |  |  |  | **H** | **L** | **L** | **L** |
| **Nuttall's Woodpecker** |  |  |  |  |  |  |  |  |  |  |  | **-2.8%** | **-7.2%** | **11.5%** | **10.9%** | **-4.5%** | **-16.4%** | **17.6%** | **25.3%** | **STABLE** |  |  |  | **H** | **L** | **L** | **L** |
| **Pileated woodpecker** | **2.5** | **3.6** | **50.5** | **26.1** | **0.6** | **0.6** |  |  |  |  |  | **24.9%** | **26.6%** | **7.5%** | **9.1%** | **-7.3%** | **21.4%** | **4.5%** | **23.3%** | **STABLE** |  |  |  | **H** | **H** | **L** | **L** |
| **Red-bellied Woodpecker** | **14.3** | **14.3** | **200** | **145.5** | **44.1** | **32.1** |  |  |  |  |  | **14.6%** | **14.9%** | **16.0%** | **18.2%** | **54.5%** | **45.8%** | **72.6%** | **58.9%** | **STABLE** |  |  |  | **H** | **L** | **L** | **L** |
| **Red-breasted Sapsucker** |  |  |  |  |  |  |  |  |  |  |  | **-5.1%** | **-17.6%** | **17.4%** | **9.5%** | **5.4%** | **-6.2%** | **24.2%** | **17.2%** | **THREATENED** |  |  |  | **H** | **L** | **L** | **L** |
| **Red-cockaded Woodpecker** |  |  |  |  |  |  |  |  |  |  | **null** | **NA** | **NA** | **70.4%** | **94.4%** | **NA** | **NA** | **32.3%** | **50.8%** | **THREATENED** |  |  |  | **H** | **H** | **L** | **L** |
| **Red-headed Woodpecker** | **10.9** | **9.3** | **200** | **200** | **62.2** | **33.2** |  |  |  |  |  | **7.4%** | **7.9%** | **8.8%** | **8.4%** | **49.3%** | **50.5%** | **59.0%** | **50.1%** | **STABLE** |  |  |  | **H** | **L** | **L** | **L** |
| **Red-naped Sapsucker** |  |  |  |  |  |  |  |  |  |  |  | **8.1%** | **-16.9%** | **58.1%** | **65.5%** | **-62.2%** | **-57.2%** | **174.7%** | **140.0%** | **ENDANGERED** |  |  |  | **H** | **L** | **H** | **L** |
| **White-headed Woodpecker** |  |  |  |  |  |  |  |  |  |  |  | **-27.3%** | **-33.5%** | **-6.1%** | **-23.5%** | **-98.1%** | **-82.0%** | **-68.9%** | **-48.9%** | **ENDANGERED** |  |  |  | **H** | **L** | **L** | **L** |
| **Williamson's Sapsucker** |  |  |  |  |  |  |  |  |  |  |  | **55.3%** | **-7.5%** | **92.8%** | **37.3%** | **-92.9%** | **-90.4%** | **-45.9%** | **-68.0%** | **ENDANGERED** |  |  |  | **H** | **H** | **L** | **L** |
| **Yellowbellied sapsucker** | **-77.2** | **-58.2** | **-70.6** | **-49.8** | **-34.5** | **-30** |  |  |  |  |  | **44.4%** | **62.0%** | **21.4%** | **24.9%** | **-30.7%** | **53.0%** | **64.5%** | **60.8%** | **THREATENED** |  |  |  | **H** | **H** | **L** | **L** |


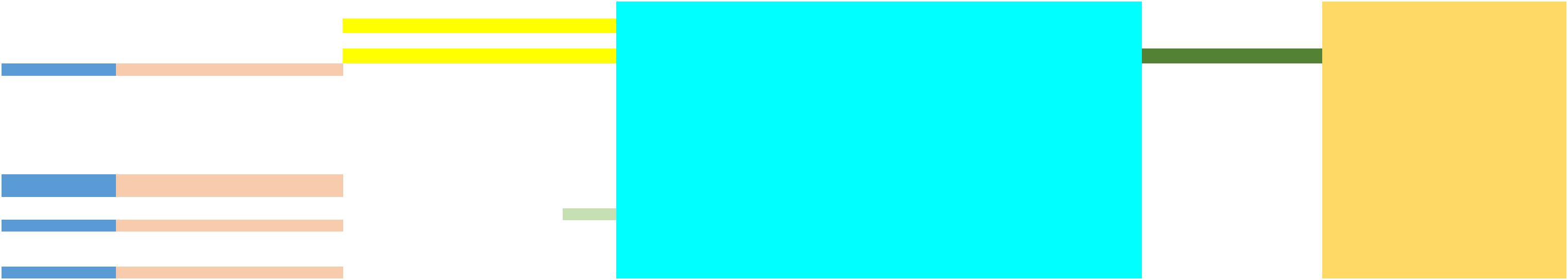


**Forecasted values represent the end of the century 2080-2100, unless otherwise noted**


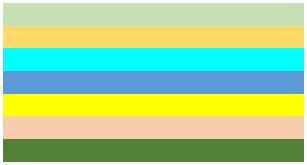


**Bancroft et al. 2016**

**Foden et al. 2013**

**Langham et al. 2015**

**Matthews et al. 2011**

**Ralston and Kirchman 2013**

**Rodenhouse et al. 2008**

**Tremblay et al. 2018**

**Table S1. The summarized woodpecker responses of the prediction studies reviewed. Only studies with reportable data are included.**
