# Supplementary material for: Climate change, woodpeckers, and forests: Current trends and future modeling needs
Source: Ecol Evol. 2019 Feb 5;9(4):2305–19. doi: 10.1002/ece3.4876 (PMC6392386; doi:10.1002/ece3.4876)
Supplement: Supplementary file 4 [file ECE3-9-2305-s004.docx]

| **Paper** | **Year** | **Season** | **Temperature** | **Precipitation** | **Climate** | **Climate** | **Bioclimatic** | **Elevation Latitude** | **Tree** | **Plant Functional Types** | **Land use** | **Land Use/** | **Trait Based** | **Survey** | **Forest Stand** | **Climatic Suitable** | **Species** | **Niche** | **Abundance** | **Demographic** | **Dispersal** | **Niche** | **Climate** | **Climate** | **Adaptive Productivity** |
| --- | --- | --- | --- | --- | --- | --- | --- | --- | --- | --- | --- | --- | --- | --- | --- | --- | --- | --- | --- | --- | --- | --- | --- | --- | --- |
|  |  |  |  |  | **Averages** | **Extremes/A** | **Variable** |  | **Species** |  | **change** | **cover** | **Climate** | **Effort** | **Characteristic** | **Habitat/Range** | **Richness** | **Flexibility** | **Response** | **Responses** | **Response** | **Temperature** | **Sensitivity** | **Exposure** | **Capacity** |
|  |  |  |  |  |  | **nomalies** | **(Aggregates)** |  |  |  |  |  | **Sensitivity** |  |  | **Distribution** |  |  |  |  |  | **Gradient** |  |  |  |
|  |  |  |  |  |  |  |  |  |  |  |  |  |  |  |  | **Response** |  |  |  |  |  |  |  |  |  |
| **Bancroft et al.** | **2016** | **B** |  | **X** |  |  |  |  |  | **X** | **X** |  |  |  |  |  |  |  |  | **X** | **X** |  |  |  |  |
| **Foden et al.** | **2013** |  | **X** | **X** | **X** |  |  |  |  |  |  |  | **X** |  |  |  |  |  |  |  |  |  | **X** | **X** | **X** |
| **Langham et al.** | **2015** | **W,B** |  |  |  |  | **X** |  |  |  |  |  |  |  |  | **X** |  |  |  |  |  |  |  |  |  |
| **Matthews et al.** | **2011** | **B** | **X** | **X** | **X** |  |  | **X** | **X** |  |  |  |  |  |  | **X** |  |  |  |  |  |  |  |  |  |
| **Ralston and Kirchman** | **2013** | **B** | **X** | **X** | **X** | **X** |  |  |  |  |  |  |  |  |  | **X** |  |  |  |  |  |  |  |  |  |
| **Rodenhouse et al.** | **2008** | **B** | **X** | **X** | **X** |  |  | **X** | **X** |  |  |  |  |  |  | **X** |  |  | **X** |  |  |  |  |  |  |
| **Tremblay et al.** | **2018** | **B** |  |  |  |  |  |  |  |  |  |  |  |  | **X** |  |  |  |  |  |  |  |  |  | **X** |


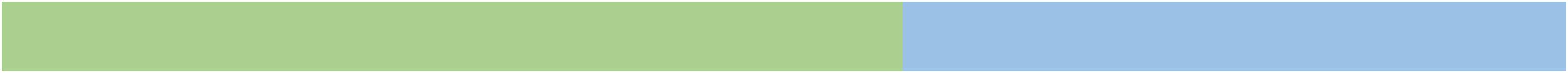

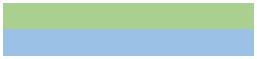


**Independent Variable**

**Response Variable**

**B, breeding season**

**W, wintering season**

**Table S2. The summarized explanatory and response variables of the predition studies reviewed.**
