# Supplementary material for: Climate change, woodpeckers, and forests: Current trends and future modeling needs
Source: Ecol Evol. 2019 Feb 5;9(4):2305–19. doi: 10.1002/ece3.4876 (PMC6392386; doi:10.1002/ece3.4876)
Supplement: Supplementary file 5 [file ECE3-9-2305-s005.docx]

| **Breeding** | **Winter** |  | **Initial laying** | **Clutch** | **Fledglings** | **Winter** | **Energetic** | **Winter** | **Breeding** | **Niche** | **Breeding** | **Breeding** | **Winter** | **Breeding** | **Breeding** | **Winter Latitude** | **Winter** | **Breeding** | **Direction of** | **Breeding** | **Breeding Upper** | **Breeding** | **Breeding** | **Climate** | **Climate** |
| --- | --- | --- | --- | --- | --- | --- | --- | --- | --- | --- | --- | --- | --- | --- | --- | --- | --- | --- | --- | --- | --- | --- | --- | --- | --- |
| **Season** | **Season** |  | **date (Julian** | **Size** | **Number** | **Community** | **Demand** | **Occupancy** | **Occupancy** | **Tracking** | **Abundance** | **Geographic** | **Northern** | **Northern** | **Latitude** | **Centroid Shift** | **Latitude** | **Southern** | **Breeding** | **Geographic** | **Elevation Range** | **Elevation** | **Lower** | **Suitability Trend** | **Suitability Trend** |
| **Studies** | **Studies** | | **Day)** |  |  | **Composition** |  |  |  |  | **Change** | **Centroid** | **Boundary of** | **Boundary of** | **Centroid** | **(occurrence)** | **Centroid Shift** | **Boundary** | **Distribution** | **area change** | **Limit Shifts** | **Centroid** | **Elevation** | **(positive)** | **(negative)** |
|  |  |  |  |  |  |  |  |  |  |  |  | **Shift** | **Range** | **Range** | **Shift** |  | **(abundance)** | **of Range** | **Shift** |  |  | **Shift** | **Range Limit** | **(Value:number** | **(Value:number** |
|  |  |  |  |  |  |  |  |  |  |  |  |  | **Latitude** | **Latitude** |  |  |  | **Latitude** |  |  |  |  | **Shifts** | **of states)** | **of states)** |
|  |  |  |  |  |  |  |  |  |  |  |  |  | **Shift** | **Shift** |  |  |  | **Shift** |  |  |  |  |  |  |  |
| **Species** |  |  |  |  |  |  |  |  |  |  |  |  |  |  |  |  |  |  |  |  |  |  |  |  |  |
| **Acorn Woodpecker** | **4** | **2** |  |  |  |  |  |  |  |  |  |  | **decrease** |  |  | **decrease** | **decrease** |  | **east** | **expanding** | **downslope** |  | **null** | **1** | **3** |
| **American Three-toed Woodpecker** | **2** | **1** |  |  |  |  |  |  |  |  | **null** | **null** |  |  |  |  |  |  |  |  |  |  |  |  | **4** |
| **Black-backed Woodpecker** | **3** | **1** |  |  |  |  |  |  | **increase** |  | **null** | **null** |  |  | **increase** |  |  | **decrease** |  |  |  | **downslope** | **upslope** |  | **11** |
| **Downy Woodpecker** | **4** | **3** |  |  |  |  |  |  |  |  | **increase** | **east** |  |  |  |  |  |  | **west** | **expanding** | **upslope** |  | **downslope** | **6** | **42** |
| **Gila Woodpecker** | **2** | **2** |  |  |  |  |  |  |  |  |  |  | **decrease** |  |  | **increase** | **decrease** |  | **northwest** | **expanding** |  |  |  | **1** |  |
| **Gilded Flicker** | **1** | **0** |  |  |  |  |  |  |  |  |  |  |  |  |  |  |  |  |  |  |  |  |  | **1** |  |
| **Golden-fronted Woodpecker** | **3** | **3** |  |  |  |  |  |  |  |  |  |  | **increase** | **null** |  | **decrease** | **decrease** |  | **west** | **no change** |  |  |  | **1** |  |
| **Hairy Woodpecker** | **5** | **2** |  |  |  |  |  |  | **null** |  | **increase** | **east** |  |  | **increase** |  |  |  | **east** | **no change** | **downslope** | **upslope** | **upslope** | **6** | **42** |
| **Ladder-backed Woodpecker** | **3** | **3** |  |  |  |  |  |  |  |  | **null** | **null** | **decrease** |  |  | **decrease** | **decrease** |  | **west** | **contracting** |  |  |  | **4** | **1** |
| **Lewis’s Woodpecker** | **2** | **2** |  |  |  |  |  |  |  |  |  |  | **decrease** |  |  | **decrease** | **decrease** |  | **north** | **no change** |  |  |  | **7** | **2** |
| **Northern Flicker** | **3** | **3** | **decrease** | **Increase** |  |  |  |  |  |  |  |  | **increase** |  |  | **increase** | **increase** |  |  |  | **null** |  | **downslope** |  |  |
| **Nuttall's Woodpecker** | **4** | **3** |  |  |  |  |  |  |  |  |  |  | **decrease** |  |  | **decrease** | **decrease** |  | **north** | **no change** | **null** |  | **null** | **1** |  |
| **Pileated Woodpecker** | **5** | **2** |  |  |  |  |  |  | **null** |  | **increase** | **northeast** |  |  | **increase** |  |  |  | **northeast** | **expanding** | **null** | **downslope** | **downslope** | **24** | **15** |
| **Red-bellied Woodpecker** | **4** | **4** |  |  |  |  |  |  | **increase** |  | **increase** | **northwest** | **increase** | **increase** | **decrease** | **increase** | **increase** |  | **northwest** | **expanding** | **upslope** | **upslope** |  | **27** | **4** |
| **Red-breasted Sapsucker** | **4** | **0** |  |  |  |  |  |  |  |  |  |  |  |  |  |  |  |  | **north** | **no change** | **null** |  | **upslope** | **2** | **1** |
| **Red-cockaded Woodpecker** | **2** | **3** | **decrease** |  | **Increase** |  |  |  |  |  |  |  | **decrease** |  |  | **decrease** | **decrease** |  |  |  |  |  |  | **4** | **2** |
| **Red-headed Woodpecker** | **3** | **2** |  |  |  |  |  |  | **decrease** |  |  |  | **decrease** | **decrease** | **decrease** | **increase** | **decrease** |  | **southwest** | **contracting** | **downslope** | **downslope** |  | **6** | **29** |
| **Red-naped Sapsucker** | **1** | **0** |  |  |  |  |  |  |  |  |  |  |  |  |  |  |  |  |  |  |  |  |  | **9** |  |
| **White-headed Woodpecker** | **4** | **2** |  |  |  |  |  |  |  |  |  |  | **increase** |  |  | **decrease** | **increase** |  | **north** | **no change** | **mixed** |  | **mixed** | **2** | **1** |
| **Williamson’s Sapsucker** | **4** | **2** |  |  |  |  |  |  |  |  |  |  | **increase** |  |  | **increase** | **increase** |  | **northeast** | **contracting** | **null** |  | **null** | **7** | **2** |
| **Yellow-bellied Sapsucker** | **4** | **4** |  |  |  |  |  |  | **increase** |  |  |  | **increase** | **decrease** | **decrease** | **increase** | **increase** | **decrease** | **east** | **expanding** |  | **downslope** | **downslope** | **1** | **10** |


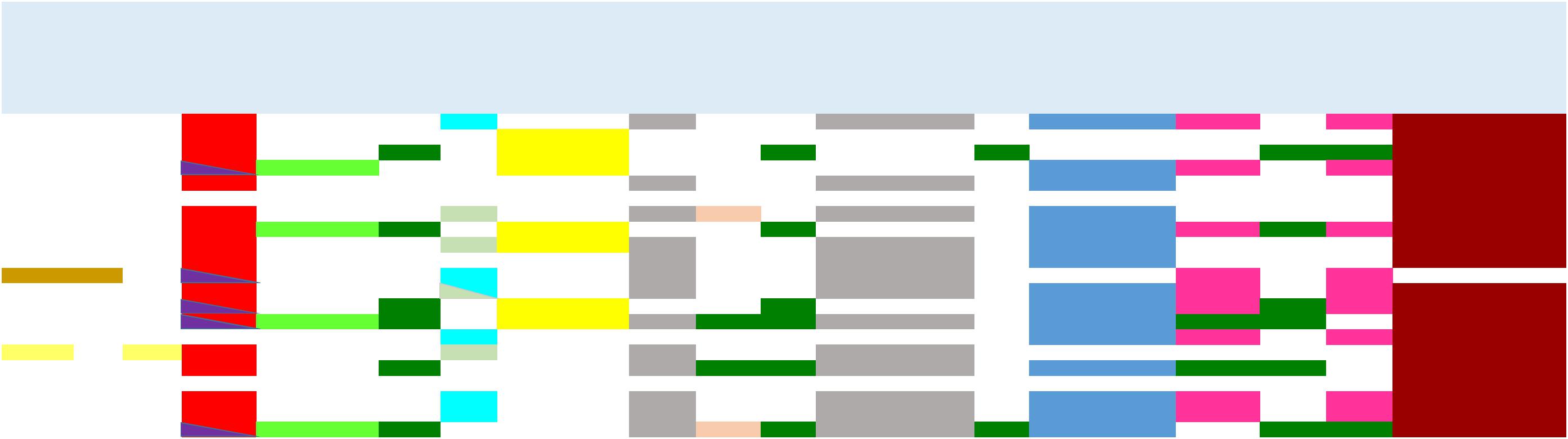

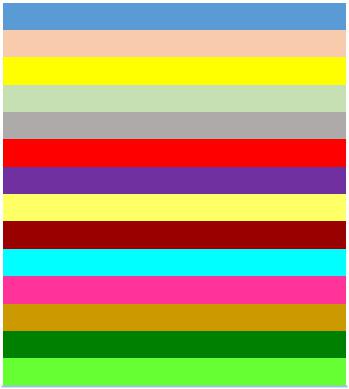


**Bateman et al. 2016**

**Hitch and Lebrg 2007**

**Huang et al. 2017**

**La Sorte and Jetz 2012**

**La Sorte and Thompson III 2007**

**La Sorte et al. 2009**

**Prince and Zuckerberg 2015**

**Schiegg et al. 2002**

**Stephens et al. 2016**

**Tingley et al. 2009**

**Tingley et al. 2012**

**Wiebe and Gerstmar 2010**

**Zuckerberg et al. 2009**

**Zuckerberg et al. 2011**

**Cells with color only (no text) denote species used in analysis. Specific responses are not able to be summarized, see respective paper(s).**

**Cells with dual colors denote multiple studies**

**Table S3. The summarized woodpecker responses of the observation studies reviewed.**
