# Supplementary material for: Climate change, woodpeckers, and forests: Current trends and future modeling needs
Source: Ecol Evol. 2019 Feb 5;9(4):2305–19. doi: 10.1002/ece3.4876 (PMC6392386; doi:10.1002/ece3.4876)
Supplement: Supplementary file 6 [file ECE3-9-2305-s006.docx]

| **Paper** | **Year** | **Temperature** | **Precipitation** | **Bioclimatic** | **Climatic** | **Climatic** | **Ratio of Actual to** | **Snow** | **Individual** | **Individual** | **Individual** | **Migratory** | **Body** | **Territory** | **Mean** | **Mean** | **Diet** | **Inbreeding** | **Land use/** | **Scale** | **Climatic** |
| --- | --- | --- | --- | --- | --- | --- | --- | --- | --- | --- | --- | --- | --- | --- | --- | --- | --- | --- | --- | --- | --- |
|  |  |  |  |  | **Average** | **extreme/a Potential** | | **depth** | **Age** | **Condition** | **Breeding** | **Strategy** | **Mass** | **Type** | **Home** | **Clutch** | **Bredth** | **Condition** | **cover** | **Dependent** | **Response was** |
|  |  |  |  |  |  | **nomaly/m** | **Evapotranspiration** |  |  |  | **Experience** |  |  |  | **Range** | **Size** |  |  |  |  | **Considered** |
|  |  |  |  |  |  | **in/max** |  |  |  |  |  |  |  |  |  |  |  |  |  |  | **Implicietly** |
| **Bateman et al.** | **2016** | **x** | **x** | **x** | **x** | **x** |  |  |  |  |  |  |  |  |  |  |  |  | **x** |  |  |
| **Hitch and Lebrg** | **2007** |  |  |  |  |  |  |  |  |  |  |  |  |  |  |  |  |  |  |  |  |
| **Huang et al.** | **2017** | **x** | **x** | **x** | **x** | **x** |  |  |  |  |  |  |  |  |  |  |  |  |  |  |  |
| **La Sorte and Jetz** | **2012** | **x** |  |  |  | **x** |  |  |  |  |  |  |  |  |  |  |  |  |  |  |  |
| **La Sorte and Thompson III** | **2007** |  |  |  |  |  |  |  |  |  |  |  |  |  |  |  |  |  |  |  | **x** |
| **La Sorte et al.** | **2009** | **x** |  |  | **x** |  |  |  |  |  |  |  |  |  |  |  |  |  | **x** |  |  |
| **Prince and Zuckerberg** | **2015** | **x** |  |  |  | **x** |  |  |  |  |  | **x** | **x** |  |  |  |  |  |  |  |  |
| **Schiegg et al.** | **2002** | **x** | **x** |  | **x** | **x** |  |  | **x** |  | **x** |  |  |  |  |  |  | **x** |  |  |  |
| **Stephens et al.** | **2016** | **x** | **x** |  | **x** | **x** | **x** |  |  |  |  |  |  |  |  |  |  |  |  |  |  |
| **Tingley et al.** | **2009** |  |  |  |  |  |  |  |  |  |  |  |  |  |  |  |  |  |  |  | **x** |
| **Tingley et al.** | **2012** | **x** | **x** |  | **x** |  |  |  |  |  |  | **x** | **x** | **x** | **x** | **x** | **x** |  |  |  |  |
| **Wiebe and Gerstmar** | **2010** | **x** |  |  |  |  |  |  | **x** | **x** |  |  |  |  |  |  |  |  |  | **x** |  |
| **Zuckerberg et al.** | **2009** |  |  |  |  |  |  |  |  |  |  |  |  |  |  |  |  |  |  |  | **x** |
| **Zuckerberg et al.** | **2011** | **x** | **x** |  |  | **x** |  | **x** |  |  |  |  |  |  |  |  |  |  |  |  |  |

**Table S4. The summarized explanatory variables of the observation studies reviewed.**
